# Supplementary material for: COVID-19 and COVID-19 vaccination experiences and perceptions among health workers during the pandemic in Ebonyi state, Nigeria: An analytical cross-sectional study
Source: PLoS One. 2024 May 9;19(5):e0303172. doi: 10.1371/journal.pone.0303172 (PMC11081237; doi:10.1371/journal.pone.0303172)
Supplement: S1 Appendix — (DOCX) [file pone.0303172.s001.docx]

**APPENDIX**

COVID-19 and COVID-19 vaccination experiences and perceptions among
health workers during the pandemic in Ebonyi state, Nigeria: an analytical cross-sectional study

The results for the dichotomized (positive and non-positive) COVID-19 and COVID-19 vaccination and the vaccination process experiences and perceptions and their predictors are presented in this appendix.

**Table of Content**

Table 1: Association between sociodemographic and background factors and the fear of getting COVID-19 2

Table 2: Association between sociodemographic and background factors and
the perceived possibility of getting COVID-19 4

Table 3: Association between sociodemographic and background factors and
the perceived importance of receiving COVID-19 vaccination 6

Table 4: Association between sociodemographic and background factors and
the fear of having severe side-effects from COVID-19 vaccination 8

Table 5: Association between sociodemographic and background factors and
the perceived protection against COVID-19 from receiving COVID-19 vaccination 10

Table 6: Association between sociodemographic and background factors and
the knowledge of COVID-19 vaccination place 12

| Table 1: Association between sociodemographic and background factors and the fear of getting COVID-19 among the 1276 study participants | | | | | | |
| --- | --- | --- | --- | --- | --- | --- |
|  | **Fear of getting COVID-19** | | **Crude results** | | **Adjusted results*** | |
|  | **Fearful^**  **809 (63.4)** | **Not fearful/not sure^^**  **n (%)**  **467 (36.6)** | cPD (97.5% CI) or  cCoef (97.5% CI) | p value | aPD (97.5% CI) or  aCoef (97.5% CI) | p value |
| Gender |  |  |  |  |  |  |
| Female | 539 (62.9) | 318 (37.1) | 0 | – | 0 | – |
| Male | 270 (64.4) | 149 (35.6) | 1.5% (-4.9–8.0) | 0.5894 | -0.8% (-6.9–5.2) | 0.7581 |
| Age, years (coefficient) | – | – | 0.5% (0.3–0.7) | <0.0001 | 0.05% (-0.4–0.5) | 0.7620 |
| Marital status |  |  |  |  |  |  |
| Not married^1^ | 330 (56.4) | 255 (43.6) | 0 | – | 0 | – |
| Married | 479 (69.3) | 212 (30.7) | 12.9% (6.9–19.0) | <0.0001 | 1.9% (-5.0–8.8) | 0.5323 |
| Educational level |  |  |  |  |  |  |
| None, primary, or secondary | 271 (49.2) | 279 (50.7) | 0 | – | 0 | – |
| Tertiary | 538 (74.1) | 188 (25.9) | 24.8% (18.8–30.8) | <0.0001 | 23.2% (16.1–30.4) | <0.0001 |
| Work category |  |  |  |  |  |  |
| Clinical staff | 696 (62.7) | 414 (37.3) | 0 | – | 0 | – |
| Non-clinical staff | 113 (68.1) | 53 (31.9) | 5.4% (-3.4–14.1) | 0.1686 | 7.8% (-1.2–16.8) | 0.0516 |
| Working experience, years (coefficient) | – | – | 0.5% (0.2–0.8) | 0.0001 | -0.1% (-0.6–0.4) | 0.6415 |
| Primary place of work |  |  |  |  |  |  |
| Private health facility^2^ | 359 (55.1) | 293 (44.9) | 0 | – | 0 | – |
| Public health facility^3^ | 450 (72.1) | 174 (27.9) | 17.1% (11.1–23.0) | <0.0001 | 9.3% (2.3–16.5) | 0.0035 |
| Level of primary place of work |  |  |  |  |  |  |
| Tertiary health facility^4^ | 176 (61.8) | 109 (38.2) | 0 | – | 0 | – |
| Primary health facility^5^ or secondary health facility^6^ | 633 (63.9) | 358 (36.1) | 2.1% (-5.2–9.4) | 0.5153 | 13.5% (4.6–22.4) | 0.0006 |
| Main source of information about COVID-19 |  |  |  | <0.0001^$^ |  | 0.0897^$^ |
| Internet, social media (whatsapp, facebook), & SMS | 81 (50.3) | 80 (49.7) | 0 | – | 0 | – |
| Traditional media (television, radio, prints) | 297 (59.2) | 205 (40.8) | 8.9% (-1.3–19.0) | 0.0497 | 10.6% (-1.7–22.8) | 0.0537 |
| Interpersonal^7^ | 431 (70.3) | 182 (29.7) | 20.0% (10.2–29.8) | <0.0001 | 4.1% (-8.9–17.4) | 0.4794 |
| Most trusted source of information about COVID-19 |  |  |  | <0.0001^$^ |  | <0.0001^$^ |
| Traditional media (television, radio, prints) | 285 (55.0) | 233 (45.0) | 0 | – | 0 | – |
| Internet, social media (whatsapp, facebook), & SMS | 65 (53.3) | 57 (46.7) | -1.7% (-13.0–9.5) | 0.7288 | 8.1% (-5.5–21.7) | 0.1814 |
| Interpersonal^7^ | 459 (72.2) | 177 (27.8) | 17.2% (10.8–23.5) | <0.0001 | 18.7% (9.6–27.9) | <0.0001 |
| Level of knowledge about COVID-19^8^ |  |  |  |  |  |  |
| Poor | 490 (66.6) | 246 (33.4) | 7.5% (1.4–13.6) | 0.0062 | 11.6% (5.4–17.8) | <0.0001 |
| Good | 319 (59.1) | 221 (40.9) | 0 | – | 0 | – |
| Level of attitude towards COVID-19 (vaccination)^9^ |  |  |  |  |  |  |
| Poor | 92 (38.5) | 147 (61.5) | 0 | – | 0 | – |
| Good | 717 (69.1) | 320 (30.9) | 30.6% (22.9–38.4) | <0.0001 | 31.0% (23.1–38.9) | <0.0001 |
| ^Very fearful or a little fearful. ^^Not fearful at all, not fearful, or not sure. cPD=Crude prevalence difference. aPD=Adjusted prevalence difference. cCoef=Crude coefficient. aCoef=Adjusted coefficient. *Adjusted for Basic knowledge of COVID-19; Attitude towards COVID-19 & COVID-19 vaccination; Source of information about COVID-19 (Main source and Most trusted source of information about COVID-19); Sociodemographic characteristics (Gender, Age, Marital status, Educational level); and Work related attributes (Work category, Years of working experience, Primary place of work (public and private), Level of primary place of work (primary, secondary, tertiary). ^$^p value of overall effect. ^1^Separated or Divorced or Widowed or Never married (Single). ^2^Patent medicine vendor (PMV), Private pharmacy, Private laboratory, Private hospital or clinic, Missionary hospital. ^3^Primary health care (PHC) centre, General hospital, Federal tertiary health centre, and Federal university teaching hospital. ^4^Federal tertiary health centre and Federal university teaching hospital. ^5^PMV, Private pharmacy, Private laboratory, Private hospital or clinic, and PHC centre. ^6^Missionary hospital and General hospital. ^7^Relatives/friends, health workers, place of work, place of worship etc. ^8^Knowledge score of <75% of the highest attainable score of 44 was poor knowledge and >=75% was good knowledge. ^9^Attitude score of <75% of the highest attainable score of 80 was poor attitude and >=75% was good attitude. | | | | | | |

The fear of getting COVID-19 and the associations between it and sociodemographic and background factors are presented in table 1. Among the 1276 study participants, 809 (63.4%) were fearful of getting COVID-19 while 467 (36.6%) were not fearful/not sure. As shown in the adjusted results, the predictors of being fearful of getting COVID-19 were: being a health worker at a primary/secondary health facility (adjusted prevalence difference (aPD) 13.5%, 97.5% CI 4.6–22.4, p=0.0006); being a health worker at a public health facility (aPD 9.3%, 2.3–16.5, p=0.0035); having a tertiary education (aPD 23.2%, 16.1–30.4, p<0.0001); good attitude towards COVID-19 (vaccination) (aPD 31.0%, 23.1–38.9, p<0.0001); poor knowledge of COVID-19 (aPD 11.6%, 5.4–17.8, p<0.0001); and the most trusted source of information about COVID-19 (adjusted p value of overall effect<0.0001).

| Table 2: Association between sociodemographic and background factors and the perceived possibility of getting COVID-19 among the 1276 study participants | | | | | | |
| --- | --- | --- | --- | --- | --- | --- |
|  | **Possibility of getting COVID-19** | | **Crude results** | | **Adjusted results*** | |
|  | **Possible^**  **n (%)**  **823 (64.5)** | **Not possible/not sure^^**  **n (%)**  **453 35.5)** | cPD (97.5% CI) or  cCoef (97.5% CI) | p value | aPD (97.5% CI) or  aCoef (97.5% CI) | p value |
| Gender |  |  |  |  |  |  |
| Female | 549 (64.1) | 308 (35.9) | 0 | – | 0 | – |
| Male | 274 (65.4) | 145 (34.6) | 1.3% (-5.0–7.7) | 0.6394 | -1.2% (-7.0–4.5) | 0.6315 |
| Age, years (coefficient) | – | – | 0.8% (0.6–1.1) | <0.0001 | 1.0% (0.6–1.4) | <0.0001 |
| Marital status |  |  |  |  |  |  |
| Not married^1^ | 328 (56.1) | 257 (43.9) | 0 | – | 0 | – |
| Married | 495 (71.6) | 196 (28.4) | 15.6% (9.6–21.6) | <0.0001 | -2.3% (-9.0–4.4) | 0.4406 |
| Educational level |  |  |  |  |  |  |
| None, primary, or secondary | 306 (55.6) | 244 (44.4) | 0 | – | 0 | – |
| Tertiary | 517 (71.2) | 209 (28.8) | 15.6% (9.5–21.6) | <0.0001 | 7.7% (1.1–14.4) | 0.0088 |
| Work category |  |  |  |  |  |  |
| Clinical staff | 712 (64.1) | 398 (35.9) | 0 | – | 0 | – |
| Non-clinical staff | 111 (66.9) | 55 (33.1) | 2.7% (-6.1–11.5) | 0.4881 | 0.1% (-8.7–8.9) | 0.9748 |
| Working experience, years (coefficient) | – | – | 1.0% (0.6–1.3) | <0.0001 | -0.3% (-0.8–0.1) | 0.1108 |
| Primary place of work |  |  |  |  |  |  |
| Private health facility^2^ | 386 (59.2) | 266 (40.8) | 0 | – | 0 | – |
| Public health facility^3^ | 437 (70.0) | 187 (30.0) | 10.8% (4.7–16.8) | <0.0001 | 0.3% (-6.3–6.9) | 0.9192 |
| Level of primary place of work |  |  |  |  |  |  |
| Tertiary health facility^4^ | 186 (65.3) | 99 (34.7) | 0 | – | 0 | – |
| Primary health facility^5^ or secondary health facility^6^ | 637 (64.3) | 354 (35.7) | -1.0% (-8.2–6.2) | 0.7588 | 2.3% (-6.4–10.9) | 0.5603 |
| Main source of information about COVID-19 |  |  |  | 0.0006^$^ |  | 0.0888^$^ |
| Internet, social media (whatsapp, facebook), & SMS | 96 (59.6) | 65 (40.4) | 0 | – | 0 | – |
| Traditional media (television, radio, prints) | 299 (59.5) | 203 (40.5) | -0.1% (-10.0–9.9) | 0.9882 | 8.9% (-2.7–20.5) | 0.0866 |
| Interpersonal^7^ | 428 (69.8) | 185 (30.2) | 10.2% (0.6–19.8) | 0.0175 | 2.1% (-10.4–14.5) | 0.7086 |
| Most trusted source of information about COVID-19 |  |  |  | <0.0001^$^ |  | <0.0001^$^ |
| Traditional media (television, radio, prints) | 283 (54.6) | 235 (45.4) | 0 | – | 0 | – |
| Internet, social media (whatsapp, facebook), & SMS | 81 (66.4) | 41 (33.6) | 11.8% (1.0–22.5) | 0.0144 | 17.9% (5.1–30.7) | 0.0017 |
| Interpersonal^7^ | 459 (72.2) | 177 (27.8) | 17.5% (11.2–23.9) | <0.0001 | 21.7% (13.2–30.2) | <0.0001 |
| Level of knowledge about COVID-19^8^ |  |  |  |  |  |  |
| Poor | 431 (58.6) | 305 (41.4) | 0 | – | 0 | – |
| Good | 392 (72.6) | 148 (27.4) | 14.0% (8.1–20.0) | <0.0001 | 6.9% (0.8–12.9) | 0.0116 |
| Level of attitude towards COVID-19 (vaccination)^9^ |  |  |  |  |  |  |
| Poor | 57 (23.9) | 182 (76.1) | 0 | – | 0 | – |
| Good | 766 (73.9) | 271 (26.1) | 50.0% (43.1–56.9) | <0.0001 | 48.4% (41.3–55.4) | <0.0001 |
| ^Highly possible or a bit possible. ^^Not possible at all, not possible, or not sure. cPD=Crude prevalence difference. aPD=Adjusted prevalence difference. cCoef=Crude coefficient. aCoef=Adjusted coefficient. *Adjusted for Basic knowledge of COVID-19; Attitude towards COVID-19 & COVID-19 vaccination; Source of information about COVID-19 (Main source and Most trusted source of information about COVID-19); Sociodemographic characteristics (Gender, Age, Marital status, Educational level); and Work related attributes (Work category, Years of working experience, Primary place of work (public and private), Level of primary place of work (primary, secondary, tertiary). ^$^p value of overall effect. ^1^Separated or Divorced or Widowed or Never married (Single). ^2^Patent medicine vendor (PMV), Private pharmacy, Private laboratory, Private hospital or clinic, Missionary hospital. ^3^Primary health care (PHC) centre, General hospital, Federal tertiary health centre, and Federal university teaching hospital. ^4^Federal tertiary health centre and Federal university teaching hospital. ^5^PMV, Private pharmacy, Private laboratory, Private hospital or clinic, and PHC centre. ^6^Missionary hospital and General hospital. ^7^Relatives/friends, health workers, place of work, place of worship etc. ^8^Knowledge score of <75% of the highest attainable score of 44 was poor knowledge and >=75% was good knowledge. ^9^Attitude score of <75% of the highest attainable score of 80 was poor attitude and >=75% was good attitude. | | | | | | |

The perceived possibility of getting COVID-19 and the associations between it and sociodemographic and background factors are presented in table 2. Among the 1276 study participants, 823 (64.5%) said it was possible for them to get COVID-19 while 453 (35.5%) said it was not possible or that they were not sure about it. The predictors of having the perception that it was possible to get COVID-19 were: having a tertiary education (aPD 7.7%, 1.1–14.4, p=0.0088); good attitude towards COVID-19 (vaccination) (aPD 48.4%, 41.3–55.4, p<0.0001); good knowledge about COVID-19 (aPD 6.9%, 0.8–12.9, p=0.0116); the most trusted source of information about COVID-19 (adjusted p value of overall effect<0.0001); and age as one year increase in age increases the probability of having the perception that it was possible to get COVID-19 by 1.0% (adjusted coefficient (aCoef) 1.0%, 97.5% CI 0.6–1.4, p<0.0001).

| Table 3: Association between sociodemographic and background factors and the perceived importance of receiving COVID-19 vaccination among the 1276 study participants | | | | | | |
| --- | --- | --- | --- | --- | --- | --- |
|  | **Important to receive COVID-19 vaccination** | | **Crude results** | | **Adjusted results*** | |
|  | **Important^**  **n (%)**  **986 (77.3)** | **Not important/ not sure^^**  **n (%)**  **290 (22.7)** | cPD (97.5% CI) or  cCoef (97.5% CI) | p value | aPD (97.5% CI) or  aCoef (97.5% CI) | p value |
| Gender |  |  |  |  |  |  |
| Female | 675 (78.8) | 182 (21.2) | 0 | – | 0 | – |
| Male | 311 (74.2) | 108 (25.8) | -4.5% (-10.3–1.2) | 0.0755 | 1.4% (-3.3–6.1) | 0.5142 |
| Age, years (coefficient) | – | – | -0.1% (-0.4–0.1) | 0.3092 | -0.3% (-0.6–0.05) | 0.0586 |
| Marital status |  |  |  |  |  |  |
| Not married^1^ | 442 (75.6) | 143 (24.4) | 0 | – | 0 | – |
| Married | 544 (78.7) | 147 (21.3) | 3.2% (-2.1–8.5) | 0.1797 | 6.8% (1.2–12.4) | 0.0061 |
| Educational level |  |  |  |  |  |  |
| None, primary, or secondary | 445 (80.9) | 105 (19.1) | 0 | – | 0 | – |
| Tertiary | 541 (74.5) | 185 (25.5) | -6.4% (-11.6–(-1.2)) | 0.0061 | -1.6% (-6.5–3.4) | 0.4804 |
| Work category |  |  |  |  |  |  |
| Clinical staff | 864 (77.8) | 246 (22.2) | 0 | – | 0 | – |
| Non-clinical staff | 122 (73.5) | 44 (26.5) | -4.3% (-12.5–3.8) | 0.2336 | 1.9% (-5.1–9.0) | 0.5365 |
| Working experience, years (coefficient) | – | – | 0.1% (-0.3–0.4) | 0.6632 | -0.1% (-0.4–0.2) | 0.6039 |
| Primary place of work |  |  |  |  |  |  |
| Private health facility^2^ | 530 (81.3) | 122 (18.7) | 0 | – | 0 | – |
| Public health facility^3^ | 456 (73.1) | 168 (26.9) | -8.2% (-13.5–(-3.0)) | 0.0005 | 6.6% (2.1–11.2) | 0.0011 |
| Level of primary place of work |  |  |  |  |  |  |
| Tertiary health facility^4^ | 139 (48.8) | 146 (51.2) | 0 | – | 0 | – |
| Primary health facility^5^ or secondary health facility^6^ | 847 (85.5) | 144 (14.5) | 36.7% (29.6–43.8) | <0.0001 | 36.1% (28.3–44.0) | <0.0001 |
| Main source of information about COVID-19 |  |  |  | 0.0391^$^ |  | 0.3520^$^ |
| Internet, social media (whatsapp, facebook), & SMS | 111 (68.9) | 50 (31.1) | 0 | – | 0 | – |
| Traditional media (television, radio, prints) | 390 (77.7) | 112 (22.3) | 8.7% (-0.4–17.9) | 0.0327 | -4.9% (-13.6–3.8) | 0.2101 |
| Interpersonal^7^ | 485 (79.1) | 128 (20.9) | 10.2% (1.2–19.1) | 0.0110 | -6.6% (-17.0–3.9) | 0.1582 |
| Most trusted source of information about COVID-19 |  |  |  | 0.0023^$^ |  | 0.0481^$^ |
| Internet, social media (whatsapp, facebook), & SMS | 79 (64.8) | 43 (35.2) | 0 | – | 0 | – |
| Traditional media (television, radio, prints) | 418 (80.7) | 100 (19.3) | 15.9% (5.5–26.4) | 0.0006 | 9.8% (0.2–19.3) | 0.0220 |
| Interpersonal^7^ | 489 (76.9) | 147 (23.1) | 12.1% (1.7–22.5) | 0.0089 | 12.1% (0.6–23.5) | 0.0179 |
| Level of knowledge about COVID-19^8^ |  |  |  |  |  |  |
| Poor | 545 (74.1) | 191 (25.9) | 0 | – | 0 | – |
| Good | 441 (81.7) | 99 (18.3) | 7.6% (2.4–12.8) | 0.0010 | 4.4% (-0.1–8.9) | 0.0287 |
| Level of attitude towards COVID-19 (vaccination)^9^ |  |  |  |  |  |  |
| Poor | 73 (30.5) | 166 (69.5) | 0 | – | 0 | – |
| Good | 913 (88.0) | 124 (12.0) | 57.5% (50.4–64.6) | <0.0001 | 51.0% (43.6–58.3) | <0.0001 |
| ^Very important or important. ^^Not important at all, not important, or not sure. cPD=Crude prevalence difference. aPD=Adjusted prevalence difference. cCoef=Crude coefficient. aCoef=Adjusted coefficient. *Adjusted for Basic knowledge of COVID-19; Attitude towards COVID-19 & COVID-19 vaccination; Source of information about COVID-19 (Main source and Most trusted source of information about COVID-19); Sociodemographic characteristics (Gender, Age, Marital status, Educational level); and Work related attributes (Work category, Years of working experience, Primary place of work (public and private), Level of primary place of work (primary, secondary, tertiary). ^$^p value of overall effect. ^1^Separated or Divorced or Widowed or Never married (Single). ^2^Patent medicine vendor (PMV), Private pharmacy, Private laboratory, Private hospital or clinic, Missionary hospital. ^3^Primary health care (PHC) centre, General hospital, Federal tertiary health centre, and Federal university teaching hospital. ^4^Federal tertiary health centre and Federal university teaching hospital. ^5^PMV, Private pharmacy, Private laboratory, Private hospital or clinic, and PHC centre. ^6^Missionary hospital and General hospital. ^7^Relatives/friends, health workers, place of work, place of worship etc. ^8^Knowledge score of <75% of the highest attainable score of 44 was poor knowledge and >=75% was good knowledge. ^9^Attitude score of <75% of the highest attainable score of 80 was poor attitude and >=75% was good attitude. | | | | | | |

The perceived importance of receiving COVID-19 vaccination and the associations between it and sociodemographic and background factors are presented in table 3. Among the 1276 study participants, 986 (77.3%) said it was important for them to receive COVID-19 vaccination while 290 (22.7%) said it was not important or that they were not sure about it. The predictors of having the perception that it was important to receive COVID-19 vaccination were: being a health worker at a primary/secondary health facility (aPD 36.1%, 28.3–44.0, p<0.0001); being a health worker at a public health facility (aPD 6.6%, 2.1–11.2, p=0.0011); being married (aPD 6.8%, 1.2–12.4, p=0.0061); and good attitude towards COVID-19 (vaccination) (aPD 51.0%, 43.6–58.3, p<0.0001).

| Table 4: Association between sociodemographic and background factors and the fear of having severe side-effects from COVID-19 vaccination among the 1276 study participants | | | | | | |
| --- | --- | --- | --- | --- | --- | --- |
|  | **Fear of having severe side-effects from**  **COVID-19 vaccination** | | **Crude results** | | **Adjusted results*** | |
|  | **Not fearful^**  **n (%)**  **603 (47.3)** | **Fearful/not sure^^**  **n (%)**  **673 (52.7)** | cPD (97.5% CI) or  cCoef (97.5% CI) | p value | aPD (97.5% CI) or  aCoef (97.5% CI) | p value |
| Gender |  |  |  |  |  |  |
| Female | 404 (47.1) | 453 (52.9) | 0 | – | 0 | – |
| Male | 199 (47.5) | 220 (52.5) | 0.4% (-6.3–7.0) | 0.9057 | -1.0% (-7.2–5.2) | 0.7170 |
| Age, years (coefficient) | – | – | 0.9% (0.6–1.1) | <0.0001 | 0.8% (0.4–1.3) | 0.0001 |
| Marital status |  |  |  |  |  |  |
| Not married^1^ | 233 (39.8) | 352 (60.2) | 0 | – | 0 | – |
| Married | 370 (53.6) | 321 (46.4) | 13.7% (7.5–19.9) | <0.0001 | 2.8% (-4.7–10.3) | 0.3953 |
| Educational level |  |  |  |  |  |  |
| None, primary, or secondary | 241 (43.8) | 309 (56.2) | 0 | – | 0 | – |
| Tertiary | 362 (49.9) | 364 (50.1) | 6.0% (-0.3–12.4) | 0.0318 | 12.2% (4.7–19.7) | 0.0003 |
| Work category |  |  |  |  |  |  |
| Clinical staff | 509 (45.9) | 601 (54.1) | 0 | – | 0 | – |
| Non-clinical staff | 94 (56.6) | 72 (43.4) | 10.8% (1.5–20.0) | 0.0091 | 18.3% (8.6–28.1) | <0.0001 |
| Working experience, years (coefficient) | – | – | 0.8% (0.4–1.2) | <0.0001 | -0.4% (-1.0–0.1) | 0.0960 |
| Primary place of work |  |  |  |  |  |  |
| Private health facility^2^ | 322 (49.4) | 330 (50.6) | 0 | – | 0 | – |
| Public health facility^3^ | 281 (45.0) | 343 (55.0) | -4.4% (-10.6–1.9) | 0.1191 | -1.1% (-8.8–6.6) | 0.7419 |
| Level of primary place of work |  |  |  |  |  |  |
| Tertiary health facility^4^ | 69 (24.2) | 216 (75.8) | 0 | – | 0 | – |
| Primary health facility^5^ or secondary health facility^6^ | 534 (53.9) | 457 (46.1) | 29.7% (23.0–36.4) | <0.0001 | 37.9% (29.2–46.7) | <0.0001 |
| Main source of information about COVID-19 |  |  |  | <0.0001^$^ |  | 0.0070^$^ |
| Internet, social media (whatsapp, facebook), & SMS | 46 (28.6) | 115 (71.4) | 0 | – | 0 | – |
| Traditional media (television, radio, prints) | 247 (49.2) | 255 (50.8) | 20.6% (11.2–30.1) | <0.0001 | 15.0% (4.2–25.9) | 0.0019 |
| Interpersonal^7^ | 310 (50.6) | 303 (49.4) | 22.0% (12.8–31.2) | <0.0001 | 7.5% (-3.4–18.3) | 0.1228 |
| Most trusted source of information about COVID-19 |  |  |  | 0.0018^$^ |  | 0.0285^$^ |
| Internet, social media (whatsapp, facebook), & SMS | 41 (33.6) | 81 (66.4) | 0 | – | 0 | – |
| Traditional media (television, radio, prints) | 242 (46.7) | 276 (53.3) | 13.1% (2.3–23.9) | 0.0064 | -6.8% (-19.1–5.6) | 0.2209 |
| Interpersonal^7^ | 320 (50.3) | 316 (49.7) | 16.7% (6.1–27.3) | 0.0004 | 4.1% (-8.3–16.5) | 0.4627 |
| Level of knowledge about COVID-19^8^ |  |  |  |  |  |  |
| Poor | 326 (44.3) | 410 (55.7) | 0 | – | 0 | – |
| Good | 277 (51.3) | 263 (48.7) | 7.0% (0.7–13.3) | 0.0132 | 10.5% (4.2–16.9) | 0.0002 |
| Level of attitude towards COVID-19 (vaccination)^9^ |  |  |  |  |  |  |
| Poor | 54 (22.6) | 185 (77.4) | 0 | – | 0 | – |
| Good | 549 (52.9) | 488 (47.1) | 30.3% (23.4–37.3) | <0.0001 | 21.8% (14.4–29.2) | <0.0001 |
| ^Not fearful at all or not fearful. ^^Very fearful, a little fearful, or not sure. cPD=Crude prevalence difference. aPD=Adjusted prevalence difference. cCoef=Crude coefficient. aCoef=Adjusted coefficient. *Adjusted for Basic knowledge of COVID-19; Attitude towards COVID-19 & COVID-19 vaccination; Source of information about COVID-19 (Main source and Most trusted source of information about COVID-19); Sociodemographic characteristics (Gender, Age, Marital status, Educational level); and Work related attributes (Work category, Years of working experience, Primary place of work (public and private), Level of primary place of work (primary, secondary, tertiary). ^$^p value of overall effect. ^1^Separated or Divorced or Widowed or Never married (Single). ^2^Patent medicine vendor (PMV), Private pharmacy, Private laboratory, Private hospital or clinic, Missionary hospital. ^3^Primary health care (PHC) centre, General hospital, Federal tertiary health centre, and Federal university teaching hospital. ^4^Federal tertiary health centre and Federal university teaching hospital. ^5^PMV, Private pharmacy, Private laboratory, Private hospital or clinic, and PHC centre. ^6^Missionary hospital and General hospital. ^7^Relatives/friends, health workers, place of work, place of worship etc. ^8^Knowledge score of <75% of the highest attainable score of 44 was poor knowledge and >=75% was good knowledge. ^9^Attitude score of <75% of the highest attainable score of 80 was poor attitude and >=75% was good attitude. | | | | | | |

The fear of having severe side-effects from COVID-19 vaccination and the associations between it and sociodemographic and background factors are presented in table 4. Among the 1276 study participants, 603 (47.3%) were not fearful of having severe side-effects from COVID-19 vaccination while 673 (52.7%) were fearful or not sure about it. The predictors of not being fearful of having severe side-effects from COVID-19 vaccination were: being a health worker at a primary/secondary health facility (aPD 37.9%, 29.2–46.7, p<0.0001); being a non-clinical health worker (aPD 18.3%, 8.6–28.1, p<0.0011); having a tertiary education (aPD 12.2%, 4.7–19.7, p=0.0003); good attitude towards COVID-19 (vaccination) (aPD 21.8%, 14.4–29.2, p<0.0001); good knowledge about COVID-19 (aPD 10.5%, 4.2–16.9, p=0.0002); the main source of information about COVID-19 (adjusted p value of overall effect=0.0070); and age as one year increase in age increases the probability of not being fearful of having severe side-effects from COVID-19 vaccination by 0.8% (aCoef 0.8%, 0.4–1.3, p=0.0001).

| Table 5: Association between sociodemographic and background factors and the perceived protection against COVID-19 from receiving COVID-19 vaccination among the 1276 study participants | | | | | | |
| --- | --- | --- | --- | --- | --- | --- |
|  | **Perceived protection from receiving COVID-19 vaccination** | | **Crude results** | | **Adjusted results*** | |
|  | **Protection^**  **n (%)**  **930 (72.9)** | **No protection/ not sure^^**  **n (%)**  **346 (27.1)** | cPD (97.5% CI) or  cCoef (97.5% CI) | p value | aPD (97.5% CI) or  aCoef (97.5% CI) | p value |
| Gender |  |  |  |  |  |  |
| Female | 629 (73.4) | 228 (26.6) | 0 | – | 0 | – |
| Male | 301 (71.8) | 118 (28.2) | -1.6% (-7.5–4.4) | 0.5591 | 3.3% (-1.8–8.4) | 0.1444 |
| Age, years (coefficient) | – | – | -0.2% (-0.4–0.06) | 0.0870 | -0.4% (-0.7–0.01) | 0.0280 |
| Marital status |  |  |  |  |  |  |
| Not married^1^ | 421 (72.0) | 164 (28.0) | 0 | – | 0 | – |
| Married | 509 (73.7) | 182 (26.3) | 1.7% (-3.9–7.3) | 0.4980 | 5.3% (-0.9–11.4) | 0.0545 |
| Educational level |  |  |  |  |  |  |
| None, primary, or secondary | 398 (72.4) | 152 (27.6) | 0 | – | 0 | – |
| Tertiary | 532 (73.3) | 194 (26.7) | 0.9% (-4.7–6.6) | 0.7164 | 7.9% (1.9–13.8) | 0.0030 |
| Work category |  |  |  |  |  |  |
| Clinical staff | 812 (73.2) | 298 (26.8) | 0 | – | 0 | – |
| Non-clinical staff | 118 (71.1) | 48 (28.9) | -2.1% (-10.5–6.4) | 0.5825 | 5.7% (-2.3–13.7) | 0.1126 |
| Working experience, years (coefficient) | – | – | -0.1% (-0.5–0.2) | 0.3683 | -0.2% (-0.6–0.3) | 0.3591 |
| Primary place of work |  |  |  |  |  |  |
| Private health facility^2^ | 494 (75.8) | 158 (24.2) | 0 | – | 0 | – |
| Public health facility^3^ | 436 (69.9) | 188 (30.1) | -5.9% (-11.5–(-0.3)) | 0.0179 | 0.9% (-5.1–7.0) | 0.7258 |
| Level of primary place of work |  |  |  |  |  |  |
| Tertiary health facility^4^ | 147 (51.6) | 138 (48.4) | 0 | – | 0 | – |
| Primary health facility^5^ or secondary health facility^6^ | 783 (79.0) | 208 (21.0) | 27.4% (20.2–34.7) | <0.0001 | 26.9% (18.6–35.3) | <0.0001 |
| Main source of information about COVID-19 |  |  |  | 0.0042^$^ |  | 0.0011^$^ |
| Traditional media (television, radio, prints) | 340 (67.7) | 162 (32.3) | 0 | – | 0 | – |
| Internet, social media (whatsapp, facebook), & SMS | 121 (75.2) | 40 (24.8) | 7.4% (-1.5–16.4) | 0.0631 | 14.8% (5.7–23.8) | 0.0003 |
| Interpersonal^7^ | 469 (76.5) | 144 (23.5) | 8.8% (2.7–14.8) | 0.0011 | 4.1% (-3.5–11.7) | 0.2319 |
| Most trusted source of information about COVID-19 |  |  |  | 0.4828^$^ |  | 0.4522^$^ |
| Internet, social media (whatsapp, facebook), & SMS | 88 (72.1) | 34 (27.9) | 0 | – | 0 | – |
| Traditional media (television, radio, prints) | 369 (71.2) | 149 (28.8) | -0.9% (-11.0–9.2) | 0.8430 | 1.8% (-8.2–11.8) | 0.6868 |
| Interpersonal^7^ | 473 (74.4) | 163 (25.6) | 2.2% (-7.7–12.1) | 0.6119 | 5.6% (-6.3–17.5) | 0.2925 |
| Level of knowledge about COVID-19^8^ |  |  |  |  |  |  |
| Poor | 526 (71.5) | 210 (28.5) | 0 | – | 0 | – |
| Good | 404 (74.8) | 136 (25.2) | 3.3% (-2.3–9.0) | 0.1811 | -2.1% (-7.3–3.0) | 0.3557 |
| Level of attitude towards COVID-19 (vaccination)^9^ |  |  |  |  |  |  |
| Poor | 61 (25.5) | 178 (74.5) | 0 | – | 0 | – |
| Good | 869 (83.8) | 168 (16.2) | 58.3% (51.5–65.1) | <0.0001 | 55.1% (47.8–62.5) | <0.0001 |
| ^Full protection or partial protection. ^^No protection at all, no protection, or not sure. cPD=Crude prevalence difference. aPD=Adjusted prevalence difference. cCoef=Crude coefficient. aCoef=Adjusted coefficient. *Adjusted for Basic knowledge of COVID-19; Attitude towards COVID-19 & COVID-19 vaccination; Source of information about COVID-19 (Main source and Most trusted source of information about COVID-19); Sociodemographic characteristics (Gender, Age, Marital status, Educational level); and Work related attributes (Work category, Years of working experience, Primary place of work (public and private), Level of primary place of work (primary, secondary, tertiary). ^$^p value of overall effect. ^1^Separated or Divorced or Widowed or Never married (Single). ^2^Patent medicine vendor (PMV), Private pharmacy, Private laboratory, Private hospital or clinic, Missionary hospital. ^3^Primary health care (PHC) centre, General hospital, Federal tertiary health centre, and Federal university teaching hospital. ^4^Federal tertiary health centre and Federal university teaching hospital. ^5^PMV, Private pharmacy, Private laboratory, Private hospital or clinic, and PHC centre. ^6^Missionary hospital and General hospital. ^7^Relatives/friends, health workers, place of work, place of worship etc. ^8^Knowledge score of <75% of the highest attainable score of 44 was poor knowledge and >=75% was good knowledge. ^9^Attitude score of <75% of the highest attainable score of 80 was poor attitude and >=75% was good attitude. | | | | | | |

The perceived protection against COVID-19 from receiving COVID-19 vaccination and the associations between it and sociodemographic and background factors are presented in table 5. Among the 1276 study participants, 930 (72.9%) said COVID-19 vaccination would give them protection against COVID-19 while 346 (27.1%) said it would give no protection or that they were not sure about it. The predictors of having the perception that COVID-19 vaccination would give protection against COVID-19 were: being a health worker at a primary/secondary health facility (aPD 26.9%, 18.6–35.3, p<0.0001); having a tertiary education (aPD 7.9%, 1.9–13.8, p=0.0030); good attitude towards COVID-19 (vaccination) (aPD 55.1%, 47.8–62.5, p<0.0001); and the main source of information about COVID-19 (adjusted p value of overall effect=0.0011);

| Table 6: Association between sociodemographic and background factors and the knowledge of COVID-19 vaccination place among the 1276 study participants | | | | | | |
| --- | --- | --- | --- | --- | --- | --- |
|  | **Knowledge of COVID-19 vaccination place** | | **Crude results** | | **Adjusted results*** | |
|  | **Knew a close place^**  **n (%)**  **927 (72.7)** | **Knew no place/ far place^^**  **n (%)**  **349 (27.3)** | cPD (97.5% CI) or  cCoef (97.5% CI) | p value | aPD (97.5% CI) or  aCoef (97.5% CI) | p value |
| Gender |  |  |  |  |  |  |
| Female | 629 (73.4) | 228 (26.6) | 0 | – | 0 | – |
| Male | 298 (71.1) | 121 (28.9) | -2.3% (-8.3–3.7) | 0.3963 | -3.6% (-9.5–2.3) | 0.1660 |
| Age, years (coefficient) | – | – | 0.5% (0.3–0.7) | <0.0001 | 0.4% (-0.06–0.8) | 0.0542 |
| Marital status |  |  |  |  |  |  |
| Not married^1^ | 392 (67.0) | 193 (33.0) | 0 | – | 0 | – |
| Married | 535 (77.4) | 156 (22.6) | 10.4% (4.8–16.0) | <0.0001 | -1.6% (-8.3–5.1) | 0.5904 |
| Educational level |  |  |  |  |  |  |
| None, primary, or secondary | 361 (65.6) | 189 (34.4) | 0 | – | 0 | – |
| Tertiary | 566 (78.0) | 160 (22.0) | 12.3% (6.6–18.0) | <0.0001 | 2.4% (-4.4–9.2) | 0.4264 |
| Work category |  |  |  |  |  |  |
| Clinical staff | 802 (72.3) | 308 (27.7) | 0 | – | 0 | – |
| Non-clinical staff | 125 (75.3) | 41 (24.7) | 3.0% (-5.0–11.1) | 0.3981 | 0.4% (-8.1–9.0) | 0.9104 |
| Working experience, years (coefficient) | – | – | 0.6% (0.3–0.9) | <0.0001 | 0.1% (-0.4–0.6) | 0.6264 |
| Primary place of work |  |  |  |  |  |  |
| Private health facility^2^ | 420 (64.4) | 232 (35.6) | 0 | – | 0 | – |
| Public health facility^3^ | 507 (81.3) | 117 (18.7) | 16.8% (11.4–22.3) | <0.0001 | 9.0% (2.1–16.0) | 0.0036 |
| Level of primary place of work |  |  |  |  |  |  |
| Tertiary health facility^4^ | 231 (81.1) | 54 (18.9) | 0 | – | 0 | – |
| Primary health facility^5^ or secondary health facility^6^ | 696 (70.2) | 295 (29.8) | -10.8% (-17.0–(-4.7)) | 0.0001 | -4.0% (-11.7–3.6) | 0.2373 |
| Main source of information about COVID-19 |  |  |  | 0.0005^$^ |  | 0.6412^$^ |
| Internet, social media (whatsapp, facebook), & SMS | 112 (69.6) | 49 (30.4) | 0 | – | 0 | – |
| Traditional media (television, radio, prints) | 337 (67.5) | 163 (32.5) | -2.0% (-11.4–7.3) | 0.6269 | 0.7% (-11.4–12.8) | 0.8952 |
| Interpersonal^7^ | 476 (77.7) | 137 (22.3) | 8.1% (-0.9–17.0) | 0.0432 | 4.1% (-8.8–16.9) | 0.4796 |
| Most trusted source of information about COVID-19 |  |  |  | <0.0001^$^ |  | 0.0574^$^ |
| Internet, social media (whatsapp, facebook), & SMS | 85 (69.7) | 37 (30.3) | 0 | – | 0 | – |
| Traditional media (television, radio, prints) | 342 (66.0) | 176 (34.0) | -3.6% (-14.1–6.8) | 0.4331 | -0.3% (-13.8–13.2) | 0.9632 |
| Interpersonal^7^ | 500 (78.6) | 136 (21.4) | 8.9% (-1.1–19.0) | 0.0454 | 8.7% (-5.2–22.6) | 0.1609 |
| Level of knowledge about COVID-19^8^ |  |  |  |  |  |  |
| Poor | 487 (66.2) | 249 (33.8) | 0 | – | 0 | – |
| Good | 440 (81.5) | 100 (18.5) | 15.3% (9.9–20.7) | <0.0001 | 11.2% (5.2–17.1) | <0.0001 |
| Level of attitude towards COVID-19 (vaccination)^9^ |  |  |  |  |  |  |
| Poor | 124 (51.9) | 115 (48.1) | 0 | – | 0 | – |
| Good | 803 (77.4) | 234 (22.6) | 25.6% (17.7–33.4) | <0.0001 | 22.6% (14.8–30.3) | <0.0001 |
| ^Knew a very close place or a close place. ^^Knew no place, a very far place, or a far place. cPD=Crude prevalence difference. aPD=Adjusted prevalence difference. cCoef=Crude coefficient. aCoef=Adjusted coefficient. *Adjusted for Basic knowledge of COVID-19; Attitude towards COVID-19 & COVID-19 vaccination; Source of information about COVID-19 (Main source and Most trusted source of information about COVID-19); Sociodemographic characteristics (Gender, Age, Marital status, Educational level); and Work related attributes (Work category, Years of working experience, Primary place of work (public and private), Level of primary place of work (primary, secondary, tertiary). ^$^p value of overall effect. ^1^Separated or Divorced or Widowed or Never married (Single). ^2^Patent medicine vendor (PMV), Private pharmacy, Private laboratory, Private hospital or clinic, Missionary hospital. ^3^Primary health care (PHC) centre, General hospital, Federal tertiary health centre, and Federal university teaching hospital. ^4^Federal tertiary health centre and Federal university teaching hospital. ^5^PMV, Private pharmacy, Private laboratory, Private hospital or clinic, and PHC centre. ^6^Missionary hospital and General hospital. ^7^Relatives/friends, health workers, place of work, place of worship etc. ^8^Knowledge score of <75% of the highest attainable score of 44 was poor knowledge and >=75% was good knowledge. ^9^Attitude score of <75% of the highest attainable score of 80 was poor attitude and >=75% was good attitude. | | | | | | |

The knowledge of COVID-19 vaccination place and the associations between it and sociodemographic and background factors are presented in table 6. Among the 1276 study participants, 927 (72.7%) knew a close COVID-19 vaccination place while 349 (27.3%) knew a far place or no place. The predictors of knowing a close COVID-19 vaccination place were: being a health worker at a public health facility (aPD 9.0%, 2.1–16.0, p=0.0036); good attitude towards COVID-19 (vaccination) (aPD 22.6%, 14.8–30.3, p<0.0001); and good knowledge about COVID-19 (aPD 11.2%, 5.2–17.1, p<0.0001).
