# Supplementary material for: COVID-19 and COVID-19 vaccination experiences and perceptions among health workers during the pandemic in Ebonyi state, Nigeria: An analytical cross-sectional study
Source: PLoS One. 2024 May 9;19(5):e0303172. doi: 10.1371/journal.pone.0303172 (PMC11081237; doi:10.1371/journal.pone.0303172)
Supplement: Attachment — Submitted filename: Response to Rviewers.docx [file pone.0303172.s003.docx]

REVIEWERS’ COMMENTS AND RESEARCHERS’ RESPONSES

Thank you for your comments and for creating the time to review our manuscript.

Academic Editor

Abstract

1. **Comment:** Please summarize the background, it is too robust for an abstract.

**Response:** The background has been reduced

2. **Comment:** From my finding, the KoBoCollect, was accessed using a link through participants WhatsApp and those that didn’t have WhatsApp filled electronic questionnaire. Please, state clearly as such.

**Response:** It has been modified as appropriate (an electronic questionnaire administered via WhatsApp or KoboCollect for those that did not have WhatsApp)

3. **Comment:** Conclusion line 54-58 please, rephrase.

**Response:** It has been rephrased

Introduction

1. **Comment:** Do you mean the reduction in the use and observance of COVID-19 control measures and decrease in the COVID-19 vaccination acceptance by the public?
   Line 76-76, please rephrase.
   Line 89-92. Please rephrase.

**Response:** Yes. The statement regarding use of control measures refers to the general population which include the health workers. Both have been modified as appropriate.

2. **Comment:** The aim/objective of the study should be the last sentence in the introduction. Please, clearly state it in a sentence. Delete line 108- “and the report of the findings are presented in this paper”

**Response:** The phrase has been deleted and the statements regarding the aim has been modified as suggested.

Methods

Study design and participants

1. **Comment:** Line 112- “all categories of health workers” please, state the categories of health workers interviewed clearly. Clinical and nonclinical staff in the private health sector should be another sentence.

**Response:** We understand or believe the statement “… all categories of health workers, both clinical and non-clinical staff in public and private health care sectors, ...” is appropriately stated. The phrases “both clinical and non-clinical” and “public and private” already define the preceding phrase “all categories”. The clinical and non-clinical staff are respectively listed in the legend of table 1, including their categorization into public and private. Should the health workers still be listed in this section as they are in the legend of table 1?

2. **Comment:** Line 114-“Eligible health workers”, inclusion criteria? Clearly state your inclusion and exclusion criteria for the study.

**Response:** The eligibility criteria (which is also called inclusion and exclusion criteria) are already clearly stated. Please note that eligibility criteria can either be stated as “eligibility criteria” or separated into “inclusion and exclusion criteria”. We chose the former, and used it in the published protocol, as the latter is not always necessary and do not always add value (Reference: STROBE reporting guidelines). It will not be appropriate to separate it into the “inclusion and exclusion criteria” format at this stage.

3. **Comment:** Line 114-115 “Eligible health workers were those who work or live in Ebonyi state and gave verbal consent and they were selected by convenience and snowballing techniques”
   Selection process; ”sampling technique” applied for the study should be separated from eligibility criteria.

**Response:** We used the unstructured format to reduce the number of subheadings and word count. They have been separated into different sentences in line with the suggestion.

4. **Comment:** Line 114-115 “The study protocol is described elsewhere.7” state the link or cite the protocol that was used.

**Response:** We seem not to understand this comment because the protocol is already cited (citation 7).

5. **Comment:** Line 115-123 should be under the topic data collection

**Response:** The statements in lines 112–119 (not 115–123) of the original manuscript were meant to describe the convenience and snowballing sampling technique. Some of them also fit into the “data collection” subsection. They have been merged under one subheading in line with other subsequent comments.

6. **Comment:** Line 124 Independent factors and outcome measures; this should be under the title questionnaire as details that follow described the various sections of the questionnaire while the rest of the explanation should be part of the data analysis as it clearly described how the data from the responses were graded after analysis.
   I noticed that there are a lot of repetitions with regard to the questionnaire

**Response:** Please note that it will not be appropriate at all to use “questionnaire” as a subheading here because this section did not describe sections of the questionnaire. It rather described the relevant independent and outcome factors (regarding the aim of this study) which were created from items in the questionnaire and the explanations were descriptions of how these factors were created. For example, level of knowledge and level of attitude (as independent factors) and extent of COVID-19 experience and perception, level of COVID-19 vaccination expectation and perception, and level of COVID-19 vaccination process experience and perception (as outcome factors) were not questionnaire items. Also, the creation of these variables was done before analysis and ideally falls under “data management” and not “statistical analyses” and because of the extent of these variable creation, we believe it is better to put it under a separate subheading than “statistical analyses”. The methods section has been rearranged in response to other subsequent comments.

We do not understand what is meant by “a lot of repetitions with regard to the questionnaire” because there are no such repetitions. Please not that this section is not (and should not be seen as) description of sections of the questionnaire (which was under data collection). It is rather a description of the relevant independent and outcome factors which were created from items in the questionnaire. Also, extent/level of COVID-19 and COVID-19 vaccination (process) experiences and perceptions were a different set of outcome measures from the dichotomized positive versus non-positive categories of COVID-19 and COVID-19 vaccination (process) experiences and perceptions.

7. **Comment:** Clearly define your methodology:
   • Study site/ study area: some details on the study site and health facilities in Ebonyi State.
   • Study design
   • Inclusion and exclusion criteria
   • Sampling techniques
   • Questionnaire design
   • Ethical clearance and consent for participation
   • Data management
   o Data collection
   o Data analysis

**Response:** The structure/subheadings in the method section has been modified as suggested. However, kindly note that the modification is also: in consideration of PLOS ONE Guidelines to authors to limit manuscript sections and sub-sections to 3 heading levels, in line with our foregoing responses, and in consideration of the fact that this study was nested in a larger study with a published protocol (and the need to maintain reasonable fidelity to the protocol). We do not understand what is actually meant by “questionnaire design”. The relevant sections of the questionnaire are already stated under data collection.

Results

**Comment:** Line 276, 282- please, do not start a sentence with a figure

**Response:** There were not new sentences but part of the statements under the “colon” in the opening sentence but the “semi-colons” to indicate these were omitted. These have been corrected.

Discussion

1. **Comment:** Line 295-297 seems like the aim of the study, please delete to avoid repetition.

**Response:** It has been deleted.

2. **Comment:** Line 311-314, please refer the outcome of the study to Ebonyi State rather than generalizing it to Nigeria since the study was conducted in Ebonyi state and the method of sampling was not randomized.

**Response**: It has been modified as suggested**.**

3. **Comment:** Line 315-319 could it be generally classified as infodemic with regard to COVI-19 and COVID-19 vaccination?

**Response:** Misinformation simply refers to false information. Disinformation is a specific type of misinformation created with the intention to deceive (that is, deliberate spread of false information). Both are preferred in this context. Infodemic simply means too much of information including false information and would not properly capture what we intend to emphasize.

4. **Comment:** Limitations of the study should be the last paragraph of the discussion.
   Discuss the strength of the study before the limitations of the study.

**Response:** The order of the limitation and strength has been changed as suggested.

5. **Comment:** Line 418-420 “Hence, the study findings are more generalizable to the general population of health workers in the state, other states in Nigeria, and other poor resource settings with limited internet access.”
   The above statement mentioned that this study could be generalized to other states in Nigeria. Going by the sampling method that was used in the study could we generalize it to Nigeria? It will be more appropriate to limit our findings to Ebonyi State.

**Response:** The generalization as used in this context was in comparison to the only online studies as our study was both online and offline and thus, more representative of the general population of health workers (in urban and rural areas). However, it has been modified.

Conclusion

**Comment:** Line 438-446, this should be stated as part of the limitation of the study and also the appeal for further studies to cover the qualitative aspect of the current study. Rephrase and make it more concise to fit into part of the limitation of the study.

**Response:** We do not see this as limitation that should be placed under the “limitation” section. Such recommendation for further studies are common in relevant literature and are based on all the available evidence (from our study and other previous studies) as discussed under the “discussion” section.

Reviewer # 1

**Comment:** check your reference

**Response:** We do not understand what specific issue to check in the reference because no specific comment or suggestion was made. However, please note that we used software to manage the referencing and will crosscheck as appropriate.

Discussion

1. **Comment:** Please, discuss your results in details.

**Response:** We strongly believe the results have already been appropriately discussed in details.

2. **Comment:** Mention the limitations in study

**Response:** The limitations are already mentioned. Please see lines 413–423.

Conclusion

**Comment:** Please rewrite conclusion again to be more informative.

**Response:** We do not understand the comment. The conclusion is based on the study findings and the discussion of all available evidence.

Overall

**Comment:** I would advise that the manuscript is checked for grammatical and spelling errors

**Response:** The manuscript has been proof read again for typos and other errors

Reviewer # 2

**Comment:** I can see that a lot of work has gone into this paper, the topic is relevant and pertinent. Unfortunately my sense is that at the moment it is not sufficiently rigorously enough designed, i.e. in terms of the meanings of the responses to illustrate new knowledge. The authors may wish to use some of the clearer questions to reanalyse the data to enhance rigour.

**Response:** Please note that, as stated in the last part of the introduction section, this study was nested in a much larger study which was pre-registered before implementation and has a published study protocol which is also cited in this manuscript. As also stated in the “study strength” section, the outcomes and potential covariates were pre-specified in the study protocol. The statement “The authors may wish to use some of the clearer questions to reanalyse the data to enhance rigour” perhaps was due to lack of basic understanding of our study concepts and analyses.

Regarding the comment about new knowledge, please note the following:

(a) “New knowledge” is a spectrum and that in most cases, especially in applied disciplines/research, research evidence are not completely/absolutely new
(b) In many cases research evidence generation is incremental with subtle additions
(c) In most cases the assessment of “new knowledge” or “novelty” is context-specific
(d) Research evidence in many (perhaps in most) cases are corroborative of existing evidence and need not to be completely/absolutely new to be of scientific or public health or health policy importance as the importance of any evidence is also context-specific.

Title

**Comment:** Descriptive, appropriate, if a little long, could a few ands be omitted?

**Response:** The study is analytical, not descriptive. Please note that there should be no inferential analysis (use of test statistics) in descriptive studies which should only be “descriptive” as the name indicate.

The title is not longer than necessary considering the nature/purpose of the study. Rather, in line with STROBE guidelines, the title appropriately captures the study design and research questions in addition to the participants and study setting.

Abstract

**Comment:** It’s detailed, but could benefit from a little clarification and a few more commas in places, or shorter sentences, as per sections highlighted in yellow.

‘Background: The COVID-19 pandemic continues to be a global emergency with
millions of new infections/re-infections and thousands of related deaths occurring
weekly globally and requires long-term management/control. One of the factors
sustaining the pandemic is the reduction in the use/non-use of control measures.
Health workers continue to be at high risk of contracting COVID-19 because of their
key roles in the management/control of the new COVID-19 infections/re-infections’

State needs capital letter, needs breaking up into shorter sentences.

**Response:** Shorter sentences cannot enhance clarification because the variable names are long. There is no highlighted section in the pasted quote. However, the abstract has been modified in line with the suggestions of the academic editor.

Please note that the primary word is “state” but “State” can also be used, when referring to a country (e.g Nigeria) or part of a country (e.g Ebonyi). Writing it as “Ebonyi state” is very okay. This can easily be confirmed using standard dictionaries e.g Oxford learners dictionaries. From experience, we very much understand that many scholars, particularly in Nigeria, do not understand the usage of the word as we have explained above.

Methods, Results, Conclusion

1. **Comment:** It might be advisable to reduce the contents of the abstract, as its not clear what some of these results mean in practice, e.g. ‘strong C-19 experience and perception‘

**Response:** There are no highlighted sections in all the pasted quotes. However, the abstract has been modified in line with the suggestions of the academic editor.

Also note that, as stated in the manuscript text under the methods section, the related questionnaire items were scored and the scores were summed and categorized (dichotomized) as strong versus not strong COVID-19 experience and perception using the 50^th^ percentile as cutoff.

Introduction

**Comment:** This is repetitious, and could be shortened e.g. ‘An understanding of health workers’ COVID-19 experiences and perceptions, and the determinants, would be useful in the subsequent planning of tailored COVID-19 behaviour change communication strategies’ Could the authors say what the determinants refers to, also what behaviour change they are referring to- is it increased vaccine take-up

**Response:** Please note that due to the nature of the study and the concepts that were explored, the manuscript and manuscript sections are better understood as a whole. For example, to understand the statements in the above comment, you have to understand (and remember) all the preceding statements in the introduction.

For example, the preceding statement in lines 81–84 (of the revised manuscript) contains the behaviours being referred to subsequently in lines 97–98 and to continue to restate them will unnecessarily increase word count.

Also note that the determinants being referred to where the potential determinants the study set out to identify which could not be stated here under the introduction but rather presented as part of the findings in the result section.

Methods, Results

**Comment:** Unfortunately the categories for many of the resulting questions are too vague to add new knowledge, what does ‘close’ ‘good’ ‘poor’ mean.

**Response:** We have addressed this comment in our responses to the comments above

**Other Comments:**

At the moment this paper is not ready for publication. I'm not an expert on survey’s so perhaps I'm not the person to be reviewing here. The number of responses is very impressive, and the time and energy spent on recruitment and data analysis is impressive. However, my sense is that the questions and responses presented here are too vague to illustrate new knowledge.

If the authors can utilise and analyse some survey questions with really detailed, specific questions, and resubmit, with a clear aim, that could illustrate new knowledge, my sense is at the moment its not there.

**Response:** We have already addressed these comments in our responses to preceding comments. It seems the reviewer perhaps had no requisite experience or expertise to review this manuscript.
