# Supplementary material for: COVID-19 and COVID-19 vaccination experiences and perceptions among health workers during the pandemic in Ebonyi state, Nigeria: An analytical cross-sectional study
Source: PLoS One. 2024 May 9;19(5):e0303172. doi: 10.1371/journal.pone.0303172 (PMC11081237; doi:10.1371/journal.pone.0303172)
Supplement: Attachment — Submitted filename: Plos One Covid paper review 2024.docx [file pone.0303172.s004.docx]

Plos One Covid paper review 2024

- Title Clarity: The title is quite long and complex. Consider simplifying it for clarity while still capturing the essence of the study. For instance, "Experiences and Perceptions of COVID-19 and Vaccination Among Health Workers in Ebonyi State, Nigeria: A Cross-Sectional Analysis."

Avoid Redundancies: The title repeats "COVID-19" twice, which could be streamlined for brevity.

- It would be nice if you highlighted the implications of your findings in the abstract.
- The introduction successfully sets the stage by describing the global emergency status of the pandemic and its specific impact on Africa. This context is crucial for understanding the study's significance.
- Some sentences are lengthy and could be made more concise. Shorter, clearer sentences would improve readability.
- Page 3, Paragraph 1, Lines 60-61: The sentence beginning "Despite the successes recorded..." is quite long and complex. It could be broken down into shorter sentences for better readability.
- Page 3, Paragraph 2, Lines 72-73: The phrase "One of the factors sustaining..." is a bit vague. It might be helpful to specify which particular control measures are being referred to, and how they directly relate to the pandemic's management.
- Limitations of Methodology: While some limitations are inherent in the study design (like sampling method), it would be helpful to discuss these limitations more explicitly. This includes addressing the potential biases and constraints that might affect the interpretation of the findings.
- Study Design and Participants: The study is described as an analytical cross-sectional survey among health workers in Ebonyi State. The inclusion of both clinical and non-clinical staff is commendable for a comprehensive understanding. However, the use of convenience and snowballing sampling techniques might limit the generalizability of the findings. It’s important to discuss how this sampling approach may influence the results.
- Data Collection Process: The use of a structured electronic questionnaire, distributed via WhatsApp and KoBoCollect, is innovative and suitable for the current digital age. However, there might be a need to elaborate on how the questionnaire was developed and validated to ensure it accurately captures the required information.
- Statistical Analysis: The manuscript mentions the use of descriptive statistics, bivariate, and multivariate generalized linear models for data analysis. It would be beneficial to provide more details about the variables included in the models and the rationale for choosing these specific statistical methods.
- Limitations of Data: Acknowledge any limitations inherent in your data, such as those arising from the sampling method or response biases.
- Discussion of Key Findings: Briefly interpret the significance of these findings in the context of your research objectives.
